# Study on Computer Screening and Drug Properties of Herbs Intervening in Copper Death

**DOI:** 10.1155/2023/3311834

**Published:** 2023-01-11

**Authors:** Zhong Dayuan, Li Lan, Cheng Hui, Li Huanjie, Liu Yumei, Luo Yumiao, Liu Deliang, Li Dingxiang, Deng Yihui

**Affiliations:** ^1^Guangdong Provincial Hospital of Integrated Traditional Chinese and Western Medicine, Foshan 528200, China; ^2^Hunan University of Traditional Chinese Medicine, Changsha 410208, China; ^3^Foshan Hospital of Traditional Chinese Medicine, Foshan 528099, China; ^4^Jinan University, Guangzhou 510632, China

## Abstract

**Objective:**

The objective of this study was to explore the medicinal properties of herbal medicines that can interfere with the copper death pathway.

**Methods:**

The Human Gene Database, Chemical Interactions in Comparative Toxicogenomics Database, Encyclopedia of Traditional Chinese Medicine, China Medical Information Platform, and Cytoscape software were used to find target and chemicals that interfere with copper death targets, as well as herbal medicines containing these chemicals and their four natures and five flavors (basic properties of herbal medicines).

**Results:**

27 copper death-related targets were finally retrieved, as well as 2143 chemicals that could interfere with them, including 180 herbal compounds. The compounds with the highest degree values (number of nodes connected to this node) were folic acid, resveratrol, and quercetin. The 180 compounds were related to 278 herbs; those with the highest degree values (number of nodes connected to this node) were *Jujubae Fructus*, *Ginkgo biloba L*, and *Acanthopanax senticosus*. The 27 copper death targets were indirectly associated with 278 herbs; those with the highest degree values (number of nodes connected to this node) were *Achyranthis Bidentatae Radix*, *Polygonum cuspidatum Sieb. et Zucc*, and *Mori Folium*. Among the 278 herbs, 6 had incomplete information. A pharmacological analysis showed that among the 272 Chinese herbs, the most frequent meridians were the liver (133), lung (104), and spleen (91). Of the four natures, the most frequent were cold (73), warm (68), and flat (45). Of the five flavors, the most frequent were bitter (165), pungent (116), and sweet (99).

**Conclusion:**

This study preliminarily discussed the material basis and medicinal properties of herbs that can intervene in copper death, which can provide reference for the theoretical discussion, drug development, and clinical research of Chinese medicine regulating copper death.

## 1. Introduction

Programmed cell death (PCD) is ubiquitous in the development of organisms and is an ordered death initiated by genes. PCD includes apoptosis, pyroptosis, iron death, autophagy, and the newly discovered copper death [1, 2]. Copper is a metal element widely present in nature; it is also a necessary trace element for the human body and participates in various physiological activities such as energy metabolism, antioxidant processes, neurotransmitter formation, iron metabolism, and other important life activities [3]. The valence of copper is usually +1 or +2. The copper ingested by mammals enters the tissue fluid and plasma in the state of Cu^2+^, which is reduced to Cu^+^ by epithelial cell reductase and then bound to the cell with ceruloplasmin (CP) [4]. Copper ions play an important role in the normal function and physiological activity of copper proteins. When the copper ion content is too low, the function of copper ion-related enzymes is damaged. On the contrary, apoptosis and oxidative stress are activated, resulting in tissue and organ damage [5, 6]. Excessive copper ions can even directly lead to cell death without apoptosis, as seen in excessive accumulation of copper ions in cells to produce copper toxicity, resulting in cell death. The mechanism of copper ionophore-induced cytotoxicity has not been clearly studied. Recently, Tsvetkov et al. found that when known cell death modes (e.g., apoptosis, iron death, pyroptosis, and necrotizing apoptosis) were blocked, abnormally elevated copper ions in human cells could still induce cell death. This copper-dependent regulated cell death pattern is closely related to mitochondrial respiration. It occurs mainly through the direct combination of copper with the fatty acylation components of the tricarboxylic acid cycle, where the accumulation of fatty acylation proteins and the subsequent loss of Fe-S cluster proteins lead to protein toxicity stress and ultimately cell death [2]. This process is related to the ferredoxin 1 (FDX1), lipoic acid synthetase (LIAS), lipoyltransferase 1 (LIPT1), dihydrolipoamide dehydrogenase (DLD), dihydrolipoamide S-acetyl transferase (DLAT), pyruvate dehydrogenase E1 subunit alpha 1 (PDHA1), pyruvate dehydrogenase E1 subunit beta (PDHB), metal regulatory transcription factor 1 (MTF1), glutaminase (GLS), and cyclin-dependent kinase inhibitor 2A (CDKN2A) proteins, as well as the protein acylation mechanism. FDX1 and protein acylation are key regulators of copper death. Protein acylation is a highly conserved posttranslational modification of lysine, which regulates protein function by attaching lipoic acid groups to lysine residues of substrate proteins. Protein acylation only occurs in the important components of pyruvate dehydrogenase complexes such as dihydrothiooctamide S-succinyl transferase (DLST) and DLAT. Protein acylation modification of these enzymes is necessary for regulating and exerting enzymatic functions [7]. By knocking out FDX1, the expressions of DLAT and DLST in cells are completely lost, suggesting that FDX1 is the upstream regulator of protein acylation [2].

The biological effect of copper ions is U-shaped (nonlinear) [8], where high or low copper ion concentrations are not conducive to cell survival. Lack of copper ions will lead to the decrease of serum copper and CP levels and low immune response. Experimentally, severe copper deficiency has been shown to damage the innate and acquired immune functions of animals. Excessive copper can also cause cytotoxicity and damage to immune function [9]. Therefore, copper ions have bidirectional regulatory effects, and their metabolic stability is closely related to the normal functioning of intracellular functions [10]. Studies have shown that copper death is related to intracellular copper ion overload [2]. The stability of copper metabolism in vivo is not only related to food intake, gastrointestinal absorption, blood transport, hepatobiliary excretion, and distribution, but it involves also a series of processes such as intracellular copper uptake, distribution, storage, and efflux [11]. The dysfunction of the functional proteins and their modification and chaperones in each process will cause abnormal copper metabolism, leading to copper deficiency or copper overload disease [12, 13].

Copper in food is first taken by intestinal epithelial cells (IECs) and then bound to Cu-related proteins after entering the cells. It enters the peripheral circulation through the intestinal lateral membrane. Copper is then transported to the liver and integrated into the CP of the liver cells [4, 9]. CP is the most important copper transporter, as more than 90% of copper in blood is transported to various organs through CP [14]. When entering cells, copper needs the help of human copper transporter 1 (hCtr1) [15], which is a high-affinity Cu^+^ transporter that can transport Cu^+^ into the cytoplasm. In addition, Cu^+^ transport can also be achieved by ATPase copper transporting alpha (ATP7A) and beta (ATP7B) in peripheral and liver tissue cells [16]. Copper can be transported to the trans-Golgi network and vesicles by ATP7A. When copper is abundant, any excess is bound by antioxidant 1 copper chaperone (ATOX1) and then transferred to the bile duct membrane through the N-terminal metal binding domain of ATP7B to enter the bile and discharge out of the cells [17, 18]. Copper enters cells, binds to copper chaperones, and is assembled into functional proteins to prevent free copper ions from catalyzing the oxidation of intracellular biomolecules to produce reactive oxygen species [9]. Common copper chaperones are copper chaperone for superoxide dismutase (CCS), ATOX1, and cytochrome C oxidase copper chaperone COX17 (COX17) [19–21].

The regulation of cell death through copper ion metabolism has important strategic significance for the prevention and treatment of diseases. Chinese medicine has a history spanning more than 5000 years, and its role in improving disease has been verified by historical experience and current clinical research evidence. Traditional Chinese medicine involves many chemical components that can be associated with multiple targets and systems. Therefore, it is difficult to study the pathogenesis of traditional Chinese medicines. At present, the mechanisms of such medicines for treating diseases have not been clearly studied. However, the copper-induced cell death pathway broadens the application of traditional Chinese medicine in treating diseases. Therefore, the systematic summary of herbs that can regulate copper death and their properties and tastes has important strategic significance for the subsequent selection, theoretical discussion, and development of innovative medicines that can intervene in copper death.

## 2. Information and Methodology

### 2.1. Collection of Copper Death Targets

With “copper death” as the search term, the search function of the Human Gene Database (GeneCards) was used to retrieve copper death-related targets [22]. Relevance score refers to the correlation between proteins and related diseases, where the larger the relevance score, the stronger the correlation, and vice versa. In this study, a relevance score of >30 was used as the screening condition. Targets with a relevance score of 30 were included. Proteins related to copper death were supplemented through a literature collection and then through the STRING database and UniProt database query targets and UniProt numbers [23, 24]. Targets without information were removed.

### 2.2. Collection of Compounds That Interfere with Copper Death-Related Proteins

Information on the copper death targets was searched for through the gene search function of the Comparative Toxicogenomics Database (CTD) [25]. Chemicals that interfere with the targets were retrieved through the Chemical Interactions function. Then, the chemicals that could interfere with copper death targets were retrieved one by one from the Encyclopedia of Traditional Chinese Medicine (ETCM), and the retrieved chemicals were recorded as herbal compounds. Chemicals that could not be retrieved were excluded [26]. Then, a relationship network between the copper death targets and the herbal compounds was constructed by the Cytoscape software. The key compounds with the highest degree values (number of nodes connected to this node) were screened out through the network analysis function of Cytoscape 3.6.1.

### 2.3. Collection and Analysis of Herbal Medicines Related to Compounds

By searching the ETCM, the compounds' information was found one by one. Information about the compounds contained in herbal medicine was found using the Herbs Containing This Ingredient function. Then, a network of herbal compounds and herbal medicines was constructed by Cytoscape 3.6.1. The herbal medicines with the highest degree values (number of nodes connected to this node) were screened out by the network analysis function of Cytoscape. The information on Latin names, medicinal properties, meridian, four natures, and five flavors (https://www.dayi.org.cn/) of the herbs was searched for in the China Medical Information Platform (CMIP). Through the book *Chinese Materia Medica* [27], herbal information was supplemented, and herbal medicines that could not be found were eliminated.

## 3. Results

### 3.1. Collection of Copper Death-Related Targets

A total of 2133 copper death-related targets were found through the GeneCards database. Seventeen copper death-related targets were obtained (with relevance scores greater than 30). Ten additional targets related to copper death were added based on the latest literature published in *Nature* [2]. Thus, 27 copper death-related targets were entered, as shown in Table 1.

### 3.2. Collection of Compounds Regulating Copper Death Targets

**Figure 1:**
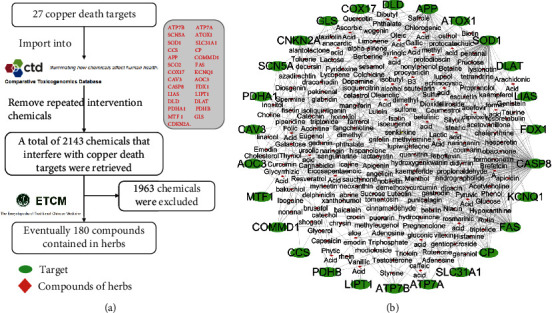
(a) Information collection process of copper death-related targets. (b) 27 relationship between copper death targets and 180 herbal compounds.

The information of the 27 copper death targets was searched for one by one through the Gene Search function of CTD. Chemicals that can interfere with the target were obtained by the Chemical Interactions function. A total of 2143 chemicals were found in the CTD database that could interfere with the 27 copper death targets, as shown in Figure 1(a). Information for these chemicals was retrieved one by one through the ETCM database, and chemicals that were not found in the database were eliminated. The results showed that the 2143 chemicals were contained in 180 herbal compounds. A relationship network between the 27 copper death targets and 180 herbal compounds was constructed by Cytoscape, as shown in Figure 1(b).

### 3.3. Screening of Core Herbal Compounds

**Figure 2:**
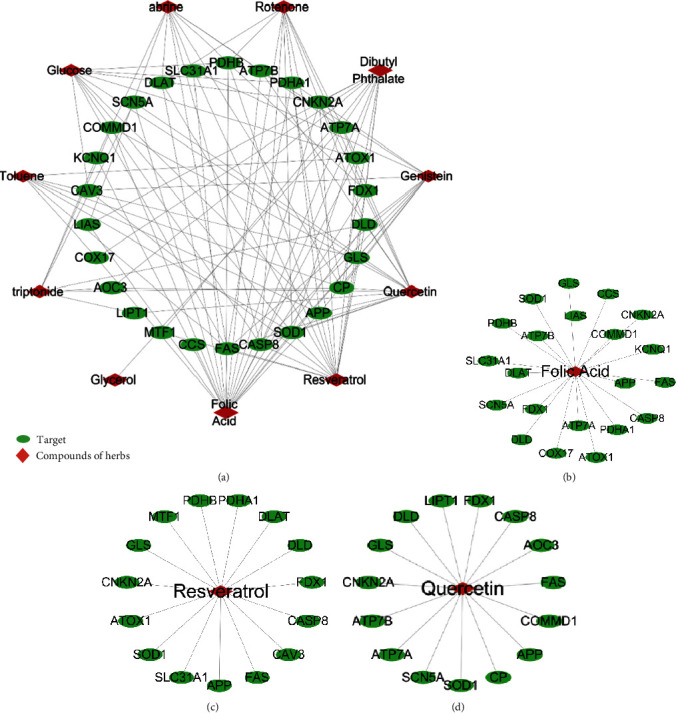
(a) Relationship network between 11 core herbal compounds and 27 targets. (b) Relationship between FA and copper death-related targets. (c) Relationship between resveratrol and copper death-related targets. (d) Relationship between quercetin and copper death-related targets.

The network analysis function of Cytoscape was used to analyze the relationship between the 27 copper death targets and 180 herbal compounds. The results showed that the 10 herbal compounds with the highest degree values (number of nodes connected to this node) were folic acid (FA), resveratrol, quercetin, genistein, dibutyl phthalate, rotenone, glucose, abrine, triptonide, and toluene. A network was constructed for these 10 core herbal compounds and their related targets, which indicated their association with 26 targets, as shown in Figure 2(a). Among them, 21 copper death-related proteins can be regulated by FA, 15 can be regulated by resveratrol, and 15 can be regulated by quercetin, as shown in Figures 2(b)–2(d).

### 3.4. Collection of Herbs Related to Herbal Compounds

**Figure 3:**
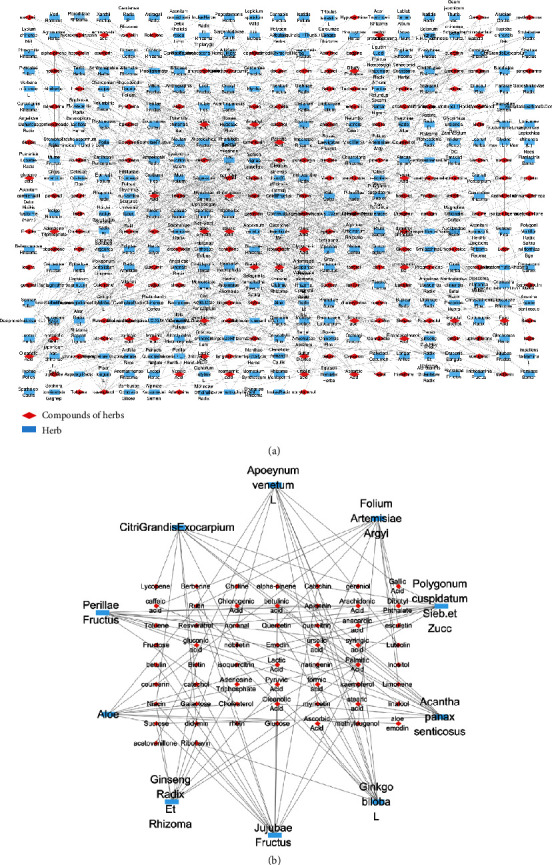
(a) 180 herbal compound and 278 herbal medicine relationship networks. (b) Relationship network of the 10 core herbs and their related herbal compounds.

The information of the abovementioned 180 herbal compounds was searched for one by one by ETCM, and 278 herbal compounds were collected. 180 herbal compound and 278 herbal medicine relationship networks were constructed by Cytoscape, as shown in Figure 3(a). An indirect network analysis was carried out on the network, and the 10 herbal compounds with the highest degree values (number of nodes connected to this node) were sorted in a descending order as follows: *Jujubae Fructus*, *Ginkgo biloba L*, *Acanthopanax senticosus*, *Polygonum cuspidatum Sieb. et Zucc*, *Folium Artemisiae Argyi*, *Apocynum venetum L*, *Perillae Fructus*, *Exocarpium Citri Grandis*, *Aloe*, and *Ginseng Radix et Rhizoma*. A relationship network of the 10 core herbs and their related herbal compounds was constructed, as shown in Figure 3(b).

### 3.5. Screening of Core Herbal Medicines

**Figure 4:**
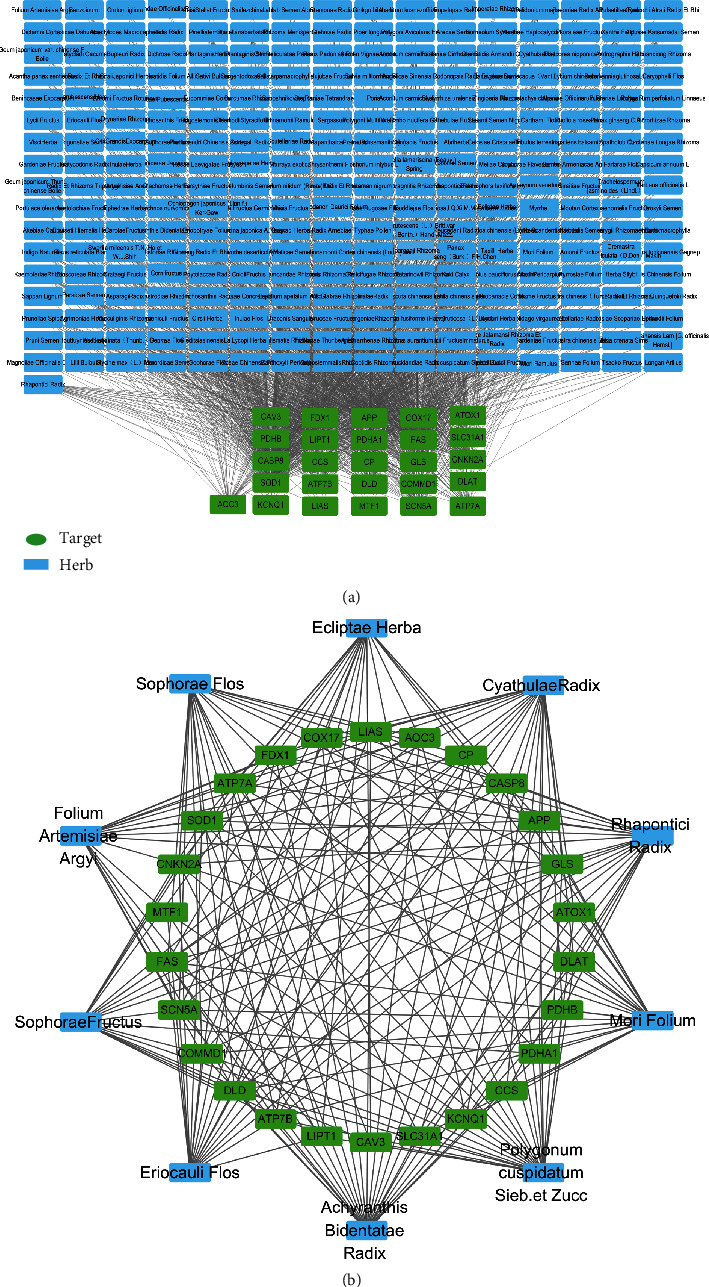
(a) Relationship networks between 27 copper death targets and 278 herbal medicines. (b) Relationship network of the 10 herbs with the highest degree values and their related copper death targets.

Relationship networks between 27 copper death targets and 278 herbal medicines were constructed by Cytoscape, as shown in Figure 4(a). An indirect network analysis was carried out on the network, and the 10 herbs with the highest degree values (number of nodes connected to this node) were sorted in a descending order as follows: *Achyranthis Bidentatae Radix*, *Polygonum cuspidatum Sieb. et Zucc*, *Mori Folium*, *Rhapontici Radix*, *Cyathulae Radix*, *Ecliptae Herba*, *Sophorae Flos*, *Folium Artemisiae Argyi*, *Sophorae Fructus*, and *Eriocauli Flos*, as shown in Figure 4(b).

### 3.6. Analysis of the Meridian Tropism, Four Flavors, and Five Flavors of the Herbs

**Figure 5:**
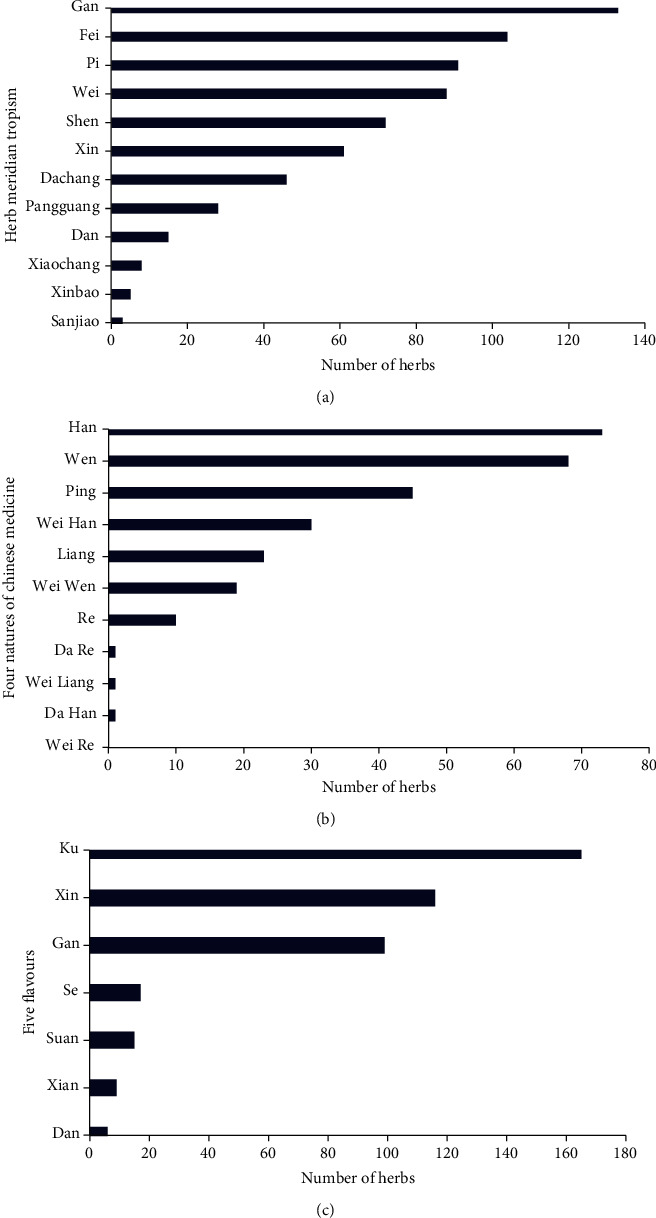
(a) Analysis results of meridian tropism of herbal medicines. (b) Analysis results of four natures of herbal medicines. (c) Analysis results of five flavors of herbal medicines.

Information about the meridian tropism, four natures, and five flavors of the herbs was obtained through the CMIP system and *Chinese Materia Medica* [27]. Information could not be found for six of the herbs. For example, the meridian tropism of *Chelidonium majus L*, *Solanum nigrum L*, *Tupistra chinensis Bak*, and *Gleditsia sinensis Lam* [*G. officinalis Hemsl*] could not be found, nor could the meridian tropism, four natures, and five flavors of *Geum japonicum* var. *chinense F. Bolle* and *Smilacina japonica A. Gray*. After the six drugs were excluded, the remaining 272 herbs were analyzed for meridian tropism, four natures, and five flavors. The results showed that the most frequent meridians found were those of the liver (133), lung (104), and spleen (91), as shown in Figure 5(a). Of the four natures, the most frequent were cold (73), warm (68), and flat (45), as shown in Figure 5(b). Of the five flavors, the most frequent were bitter (165), pungent (116), and sweet (99), as shown in Figure 5(c).

## 4. Discussion

Systematic pharmacology is a new model for studying the molecular mechanism of drugs through big data analysis [28]. By integrating multiple database resources, the process of traditional Chinese medicine research can be simplified and more accurate. By using the systematic pharmacology method, we found 180 herbal compounds that could interfere with copper death. Most of the copper death targets could be regulated by FA, resveratrol, and quercetin. FA, also known as vitamin B9, is a completely oxidized synthetic form of polyglutamate monoglutamine and a water-soluble vitamin [29]. FA is involved in many reactions, which are related to many biosynthetic pathways that allow the correct synthesis of proteins and nucleic acids in different organisms [30, 31]. So FA metabolism is essential to ensure the correct function of all living cells. Resveratrol is a kind of estradiol, a natural phenol, and plant antitoxin produced by several plants in response to injury or when plants are attacked by pathogens (e.g., bacteria or fungi). It has antioxidant, anti-inflammatory, myocardial protection, antimutation, anticancer, and other effects. It can also inhibit platelet aggregation and the activities of several deoxyribonucleic acid helicases in vitro. Quercetin is a flavonol that is widely distributed in plants and acts as an antioxidant. In this study, FA was found to regulate the expression of 21 copper death targets, including FDX1. Resveratrol can regulate the expression of 15 copper death targets, including FDX1. Similarly, quercetin can also regulate the expression of 15 copper death targets, including FDX1. In fact, current studies have shown that the regulation of FDX1 expression by these three compounds is mainly achieved by mRNA. Lin et al. found that 1,2-dimethylhydrazine and FA can lower FDX1 mRNA expression [32], as can resveratrol, according to Sadi et al. [33], and quercetin, according to Cormier et al. [34]. The important role of FDX1 in the regulation of copper death suggests that these three compounds have regulatory effects.

Through systematic pharmacological methods, we found 278 herbs containing compounds that can regulate copper death proteins. Among them, *Jujubae Fructus*, *Ginkgo biloba L*, and *Acanthopanax senticosus* contained the most compounds that could regulate copper death proteins. *Jujubae Fructus* is the mature fruit of *Ziziphus jujuba Mill*. *Ginkgo biloba L* is the seed of *Ginkgo biloba L*. Both of these two herbs have great medicinal and nutritional value and are currently recommended by the Ministry of Health of China as “dual-use resources.” Modern studies have found that *Jujubae Fructus*, which has important applications in clinical medicine, contains abundant nutrients, various trace elements, and a large number of nucleotide derivatives. Clinical studies have shown that *Jujubae Fructus* has a good adjuvant therapeutic effect on tumors, hypertension, hypercholesterolemia, and other diseases. The research and development of compounds in *Ginkgo biloba L* have mainly focused on flavonoids, terpene lactones, phenolic acids, polysaccharides, and other components. Modern studies have shown that *Jujubae Fructus* has the effects of antiaging, antioxidation, improving immunity, and liver protection [35]. *Ginkgo biloba L* has antioxidant, anti-inflammatory, neuroprotective, antitumor, antibacterial, and other pharmacological activities [36]. Whether these effects are related to the copper death pathway is unknown. By integrating traditional Chinese medicine-compound and compound-copper death target networks, we found that *Achyranthis Bidentatae Radix*, *Polygonum cuspidatum Sieb. et Zucc*, and *Mori Folium* could interfere with the most targets related to copper death. The reason may be that *Achyranthis Bidentatae Radix* contains FA compounds. *Polygonum cuspidatum Sieb .et Zucc* contains two compounds, resveratrol and quercetin. *Mori Folium* contains FA compounds. FA, resveratrol, and quercetin could regulate the most copper death-related targets. Therefore, the study of *Achyranthis Bidentatae Radix*, *Polygonum cuspidatum Sieb. et Zucc*, and *Mori Folium* regulating copper death can start with these three compounds.

This study found that most herbs that can regulate copper death are liver, lung, and spleen meridians. Of the four natures, the most frequent are cold, warm, and peaceful. Of the five flavors, the most frequent are bitter, pungent and sweet. Our previous studies have found that the meridian tropism of herbal medicine may be related to the expression differences of regulated targets in different organs [37, 38]. Considering that the number of adjustable targets of herbs is far more than the number of copper death targets, it is difficult to clarify the relationship between copper death-related targets and meridian attribution. Traditional Chinese medicine believes that the liver is the “basis of regulation” of the human body, which is similar to the neurohumoral regulation function in modern medicine [39]. Current studies on copper metabolism suggest that CP is very important in copper metabolism. As the most important copper transporter, more than 90% of copper in blood is transported to various organs via CP. CP is produced by liver cells [9], which may be the reason for the association between liver meridian and copper death. However, there are differences in the understanding of Chinese and Western medicine, particularly that of organs, as both approaches are not completely unified [40]. The relationship between liver meridian and copper death still needs a lot of rigorous experiments to study and verify.

## 5. Limitations

The data in this study are obtained from databases, lacking experimental data support. Compounds regulating copper death targets have been supported by experiments, but these studies are few and not comprehensive. It is not clear whether compounds directly bind to copper death targets or act upstream or downstream. Moreover, this study only considered the relationship and quantity among targets, compounds, and herbs. Multiple influencing factors have not been thoroughly analyzed, such as the strength of efficacy between compounds and targets, the content of compounds in different Chinese medicines, the effect of different origins on the content of compounds contained in herbs, and the effect of different boiling methods on Chinese medicine compounds. The information of the medicinal properties of some herbs is missing. Information on the medicinal properties of the herbs was collected but not statistically analyzed, and it is unclear whether there are differences in the medicinal properties of the herbs that crudely interfere with copper death.

## 6. The Direction of Future Research

Based on copper death-related proteins, this study screened 2143 factors that could interfere with 27 copper death-related proteins by searching multiple databases. Then, we searched the information of intervention factors one by one through the ETCM database and finally obtained 180 herbal compounds that could interfere with copper death-related proteins and found herb information containing the compound through the compound information. However, there are two core steps involved in this process. One is to determine that copper death-related proteins can be intervened by herbal compounds. Another is to identify the herbal compound associated with the herb. However, it is not sufficient to determine the strength of the evidence for these two steps solely in the form of a database. Future experimental studies should therefore be carried out to provide evidence that copper death-associated proteins can be intervened by herbal compounds and that herbal compounds are herb-related, for example, the category of chemical substances contained in each herb, the detection of content, bioavailability, and other related experiments. Studies on the relationship between each compound and copper death-associated protein content have included in vivo and in vitro experiments. It is also necessary to understand the mechanism of each compound interfering with copper death-related proteins, including verifying the upstream and downstream molecules of copper death-related proteins.

## 7. Conclusion

In summary, this study systematically explored the herbal compounds and the potential herbs that can regulate the mechanism of copper death from the view of copper death-related targets by systematic pharmacological methods and discussed their medicinal properties. However, the results of this study still need further experiments for verification.

## Figures and Tables

**Table 1:** Targets related to copper death.

Gene	UniProt number	Description	Source
ATP7B	P35670	ATPase copper transporting beta	a
ATP7A	Q04656	ATPase copper transporting alpha	a
SCN5A	Q14524	Sodium voltage-gated channel alpha subunit 5	a
ATOX1	O00244	Antioxidant 1 copper chaperone	a
SOD1	P00441	Superoxide dismutase 1	a
SLC31A1	O15431	Solute carrier family 31 member 1	a
CCS	O14618	Copper chaperone for superoxide dismutase	a
CP	P00450	Ceruloplasmin	a
APP	P05067	Amyloid Beta precursor protein	a
COMMD1	Q8N668	Copper metabolism domain containing 1	a
SCO2	O43819	Synthesis of cytochrome C oxidase 2	a
FAS	P25445	Fas cell surface death receptor	a
COX17	Q14061	Cytochrome C oxidase copper chaperone COX17	a
KCNQ1	P51787	Potassium voltage-gated channel subfamily Q member 1	a
CAV3	O43497	Caveolin 3	a
AOC3	Q16853	Amine oxidase copper containing 3	a
CASP8	Q14790	Caspase 8	a
FDX1	P10109	Ferredoxin 1	b
LIAS	O43766	Lipoic acid synthetase	b
LIPT1	Q9Y234	Lipoyltransferase 1	b
DLD	P09622	Dihydrolipoamide dehydrogenase	b
DLAT	A0A2K6T1J0	Dihydrolipoamide S-acetyltransferase	b
PDHA1	P08559	Pyruvate dehydrogenase E1 subunit alpha 1	b
PDHB	P11177	Pyruvate dehydrogenase E1 subunit beta	b
MTF1	Q14872	Metal regulatory transcription factor 1	b
GLS	O94925	Glutaminase	b
CDKN2A	P42771	Cyclin dependent kinase inhibitor 2A	b

Note: a means data collected from the GeneCards database; b means data collected from the article [2].

## Data Availability

Requests for additional data may be granted upon reasonable request by contacting the author (Dayuan Zhong, 13751728424@163.com).

## Abbreviations

AOC3: Amine oxidase copper containing 3
APP: Amyloid beta precursor protein
ATOX1: Antioxidant 1 copper chaperone
ATP7A: ATPase copper transporting alpha
ATP7B: ATPase copper transporting beta
CASP8: Caspase 8
CAV3: Caveolin 3
CCS: Copper chaperone for superoxide dismutase
CDKN2A: Cyclin-dependent kinase inhibitor 2A
CMIP: China Medical Information Platform
COMMD1: Copper metabolism domain containing 1
COX17: Cytochrome C oxidase copper chaperone COX17
CP: Ceruloplasmin
CTD: Comparative Toxicogenomics Database
DLAT: Dihydrolipoamide S-acetyl transferase
DLD: Dihydrolipoamide dehydrogenase
DLST: Dihydrothiooctamide S-succinyl transferase
ETCM: Encyclopedia of Traditional Chinese Medicine
FA: Folic acid
FAS: Fas cell surface death receptor
FDX1: Ferredoxin 1
GeneCards: Human Gene Database
GLS: Glutaminase
hCtr1: Human copper transporter 1
IECs: Intestinal epithelial cells
KCNQ1: Potassium voltage-gated channel subfamily Q member 1
LIAS: Lipoic acid synthetase
LIPT1: Lipoyltransferase 1
MTF1: Metal regulatory transcription factor 1
PCD: Programmed cell death
PDHA1: Pyruvate dehydrogenase E1 subunit alpha 1
PDHB: Pyruvate dehydrogenase E1 subunit beta
SCN5A: Sodium voltage-gated channel alpha subunit 5
SCO2: Synthesis of cytochrome C oxidase 2
SLC31A1: Solute carrier family 31 member 1
SOD1: Superoxide dismutase 1.
